# High-efficiency self-focusing metamaterial grating coupler in silicon nitride with amorphous silicon overlay

**DOI:** 10.1038/s41598-024-62336-0

**Published:** 2024-05-22

**Authors:** William Fraser, Daniel Benedikovic, Radovan Korcek, Maziyar Milanizadeh, Dan-Xia Xu, Jens H. Schmid, Pavel Cheben, Winnie N. Ye

**Affiliations:** 1 Silicon Micro/NanoPhotonics Group, Carleton University, Ottawa, Canada; 2 National Research Council, Ottawa, Canada; 3 Department of Multimedia and Information-Communication Technologies, University of Žilina, Žilina, Slovakia; 4 University Science Park, University of Žilina, Žilina, Slovakia

**Keywords:** Silicon photonics, Metamaterials, Sub-wavelength optics, Integrated optics

## Abstract

Efficient fiber-chip coupling interfaces are critically important for integrated photonics. Since surface gratings diffract optical signals vertically out of the chip, these couplers can be placed anywhere in the circuit allowing for wafer-scale testing. While state-of-the-art grating couplers have been developed for silicon-on-insulator (SOI) waveguides, the moderate index contrast of silicon nitride (SiN) presents an outstanding challenge for implementing efficient surface grating couplers on this platform. Due to the reduced grating strength, a longer structure is required to radiate the light from the chip which produces a diffracted field that is too wide to couple into the fiber. In this work, we present a novel grating coupler architecture for silicon nitride photonic integrated circuits that utilizes an amorphous silicon (α-Si) overlay. The high refractive index of the α-Si overlay breaks the coupler’s vertical symmetry which increases the directionality. We implement subwavelength metamaterial apodization to optimize the overlap of the diffracted field with the optical fiber Gaussian mode profile. Furthermore, the phase of the diffracted beam is engineered to focalize the field into an SMF-28 optical fiber placed 55 µm above the surface of the chip. The coupler was designed using rigorous three-dimensional (3D) finite-difference time-domain (FDTD) simulations supported by genetic algorithm optimization. Our grating coupler has a footprint of 26.8 × 32.7 µm^2^ and operates in the *O*-band centered at 1.31 μm. It achieves a high directionality of 85% and a field overlap of 90% with a target fiber mode size of 9.2 µm at the focal plane. Our simulations predict a peak coupling efficiency of − 1.3 dB with a 1-dB bandwidth of 31 nm. The α-Si/SiN grating architecture presented in this work enables the development of compact and efficient optical interfaces for SiN integrated photonics circuits with applications including optical communications, sensing, and quantum photonics.

## Introduction

Compatibility with high-yield and low-cost silicon microelectronics fabrication practices has established the silicon-on-insulator (SOI) platform as the standard for photonic integration. While the high-index contrast of SOI enables the realization of ultra-compact and high-performance optical devices^1,2^, it also increases scattering which in turn results in higher propagation losses and increased sensitivity to fabrication errors. The silicon nitride (SiN) platform has emerged as a complementary alternative to SOI. It can be paired with SOI for multi-layer monolithic circuit integration^3–5^, while helping to reduce propagation losses^6^ and advance heterogeneous integration^7^. The transparency window of silicon nitride extends down to approximately 400 nm, allowing for applications in the visible spectrum. The low refractive index contrast enhances robustness to fabrication imperfections and has led to the demonstration of ultra-low propagation losses^8^. State-of-the-art photonic structures and devices have been demonstrated on the SiN platform, including waveguides^9^, filters^10^, splitters^11^, ring resonators^12^ and on- and off-chip waveguide couplers^13^, among others.

Despite these important advances in SiN photonics, the development of efficient fiber-chip coupling strategies continues to present a challenge for the wide adoption of the platform. Sub-decibel (< 1 dB) coupling efficiency can be obtained with a variety of SiN-assisted edge couplers^13^. However, they are restricted to the perimeter of the chip which limits layout flexibility when designing photonic integrated circuits (PICs). This also makes them unsuitable for wafer-scale testing of PICs as they can only be accessed after dicing and polishing. In contrast, surface gratings can be placed anywhere on the chip, enabling flexible access to the circuit for rapid die characterization in large volumes. However, the moderate refractive index contrast between silicon nitride and the SiO_2_ cladding translates to low grating strengths. The coupling efficiency is limited by poor overlap of the radiated field with the mode of the optical fiber because a grating substantially longer than the mode field diameter (MFD) is required to diffract the light from the chip. As a result, the efficiency of SiN grating couplers is typically in the − 12 dB to − 2 dB range^14–18^. To improve the coupling efficiency between the optical fibers and SiN chips, the grating directionality needs to be enhanced and the mismatch between the radiated grating beam and the near-Gaussian fiber mode must be reduced. Gratings with single- or multi-layer bottom reflectors have been demonstrated to reduce substrate leakage, typically in the range of 20% to 30%^19–24^. However, such couplers require dedicated backside chip processing, adding complexity to the fabrication process. Alternatively, blazed SiN grating couplers with intrinsically high directionality have been reported, both theoretically and experimentally ^25,26^. These gratings leverage bi-level etching topologies or multi-layer gratings formed by SiN^27^, Si^28,29^, or SOI^30,31^ material stacks. To reach the sub-dB loss level, both near-field apodization and the self-imagining effect have been utilized to improve the fiber-to-grating field overlap^25,26,32^. These demand precise alignment between multiple patterning layers^25,26^ or a customized fabrication flow not typically available in public photonic foundries^32^. Recently, the use of high-index overlays has been shown to improve the radiation characteristics of SiN couplers and enhance the overall fiber-chip coupling efficiency^28,33–37^. To date, a variety of design strategies for hybrid α-Si/SiN grating couplers has been developed. This includes multi-layer grating configurations^28,29,33^, mirror-based couplers^34,36^, or structures based on an inter-layer mode interference effect^35^. For 800 nm thick SiN waveguides, a technique has been shown to produce a nearly Gaussian radiation profile through multi-mode excitation^34^. However, this approach is not suitable for thinner waveguides like the 400 nm SiN platform employed in our work. Alternative solutions either suffer from limited coupling performance due to poor mode field overlap or require complex fabrication processes to form backside mirrors and alleviate tensile stress defects associated with thick SiN films^4^.

In this work, we present the design of a highly efficient grating coupler for silicon nitride PICs based on a hybrid α-Si/SiN platform. Our device is optimized for the transverse electric (TE) polarization and standard SMF-28 single mode optical fibers at an operating wavelength of 1.31 µm. The grating coupler is implemented in an α-Si overlayer and can be fabricated in a single-etch process. The utilization of α-Si addresses the inherent limitations of regular silicon nitride grating couplers. By leveraging its high refractive index to augment the grating contrast and break the vertical symmetry, we substantially increase the grating strength and directionality. We further improve the coupling efficiency through subwavelength grating (SWG) metamaterial apodization to maximize the overlap with the targeted mode of the fiber^38–40^. Since the introduction of SWG metamaterials to integrated photonics^41–45^, they have been successfully used as a powerful engineering tool for overcoming performance limitations of conventional integrated photonic devices^46–48^. Finally, our design leverages a self-imaging effect to focalize the diffracted beam into an optical fiber positioned at a focal distance above the chip surface. This relaxes the constraint on grating length since the grating near-field can substantially exceed the mode field diameter (MFD) of the optical fiber mode (MFD of 9.2 µm for SMF-28 fiber at 1.31 µm), while achieving large field overlap at the focal plane. By combining a high-index overlay with SWG apodization and the self-imaging effect, our optimized design yields a coupling efficiency as high as − 1.3 dB.

### Hybrid nanophotonic platform and design methodology

**Figure 1:**
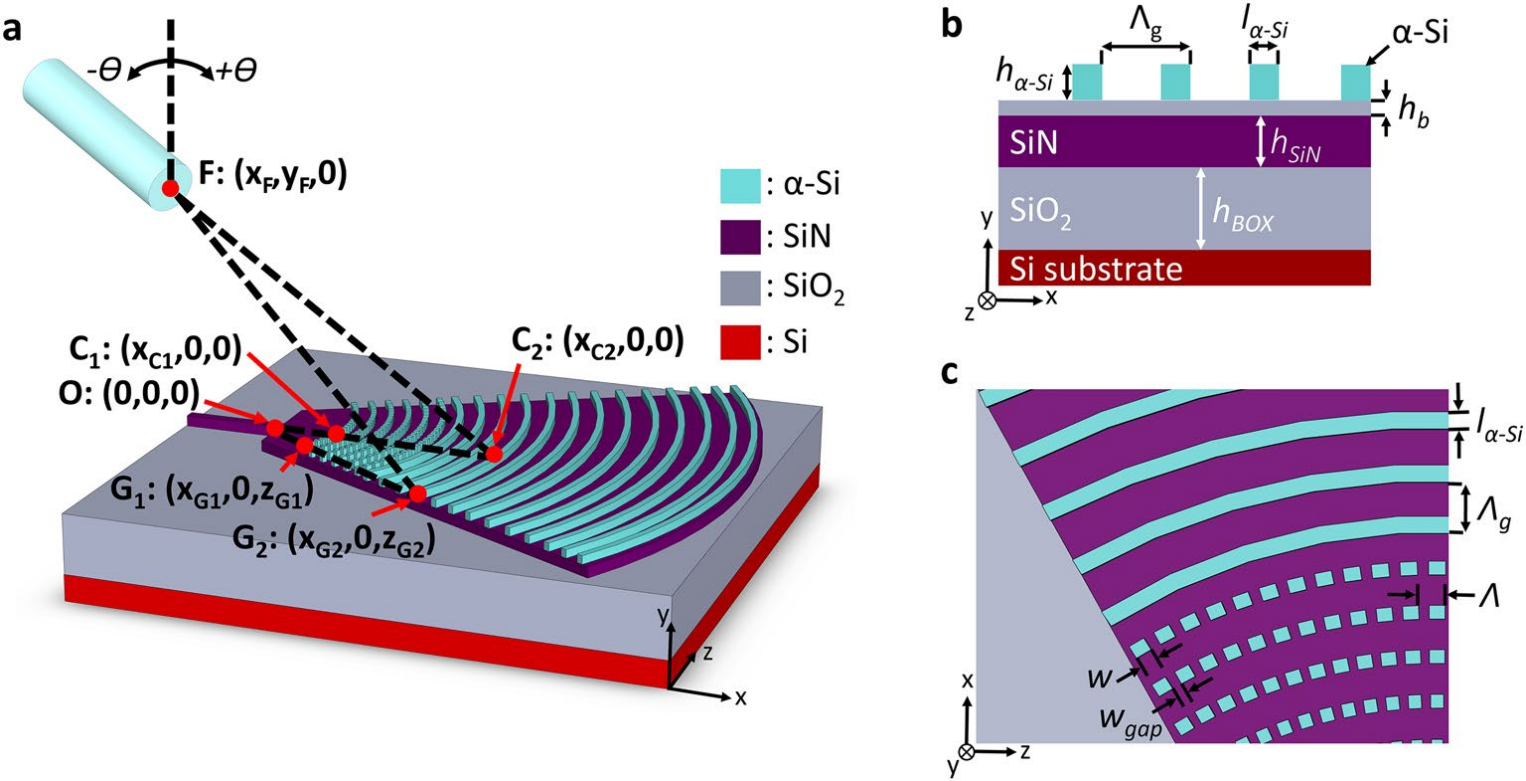
Schematics of the subwavelength metamaterial surface grating coupler on the α-Si/SiN platform. (**a**) 3D view with labelled coordinates used in calculation of grating curvature; the SiO_2_ buffer layer is transparent (not shown in (**a**), to ease visualization. (**b**) Side (x–*y* plane) and (**c**) top (*x–z* plane) views of the structure.

Our proposed grating coupler is designed for a hybrid nanophotonic waveguide platform compatible with complementary metal-oxide-semiconductor (CMOS) fabrication practices. The platform is based on a standard low-pressure chemical vapour deposition (LPCVD) SiN wafer^49^ with an amorphous silicon (α-Si) top layer. The grating is formed in the α-Si layer and can be fabricated with a single-step full etch process. A three-dimensional (3D) schematic of the surface grating coupler is illustrated in Fig. 1a. The coordinates labelled in Fig. 1a were used to derive the curvature of the grating that performs the transverse focusing required for the self-imaging effect, with the vertical *y*_f_ coordinate corresponding to the height of the fiber above the chip plane. The cross-section of the coupler is shown in Fig. 1b. At the 1.31 µm design wavelength, the SiN layer with thickness, *h*_*SiN*_, of 400 nm has a refractive index, *n*_*SiN*_, of 2.0017 according to material data sheets provided by Applied Nanotools^49^. The buried oxide (BOX) layer has a refractive index, *n*_*BOX*_, of 1.4467^50^ and a thickness, *h*_*BOX*_, of 4.5 μm. The α-Si layer of thickness *h*_*α-Si*_ is situated on top of SiN platform, separated by a buffer oxide layer of thickness *h*_b_ Both the α-Si and oxide buffer layers provide an additional degree of design freedom to optimize the grating coupler performance^28,34,35^. The respective refractive indexes are *n*_α-Si_ = 3.5187^50^ and *n*_b_ = 1.4502^49^. The superstrate is air (*n*_*c*_ = 1). The off-chip waveguide coupler is optimized for the transverse electric (TE) polarization at a nominal wavelength of 1.31 μm, typically used for applications in datacom^51,52^ and quantum^53–56^. The grating coupler was designed using full-vectorial FDTD simulations.

## Results and discussion

### Uniform surface grating coupler

**Figure 2:**
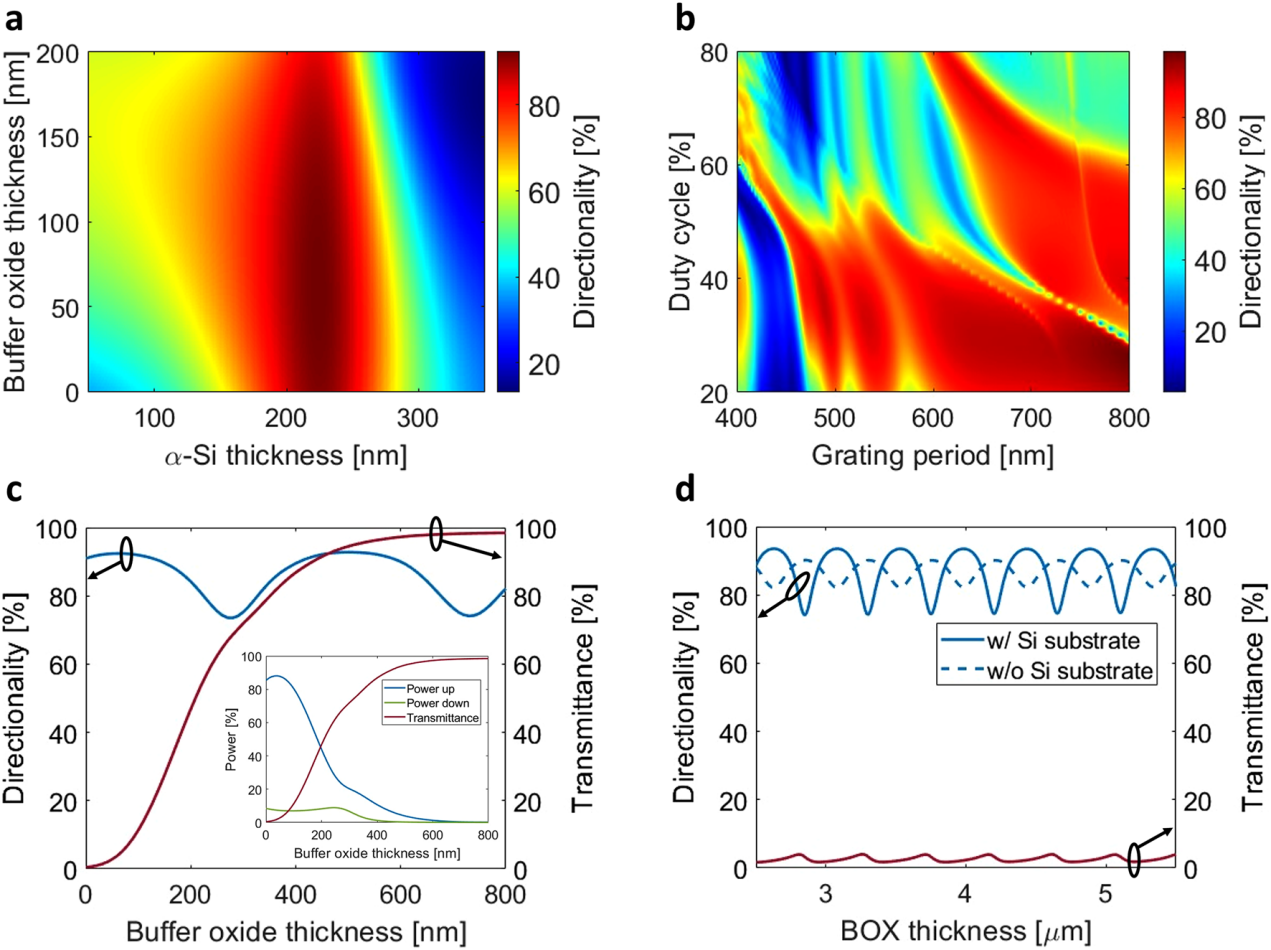
Grating directionality as a function of the (**a**) α-Si and oxide buffer thicknesses and (**b**) grating period and duty cycle. Grating directionality and forward transmittance as a function of the (**c**) buffer oxide thickness and (**d**) BOX thickness.

In the first step, we designed a uniform surface grating coupler without SWG nanostructures by performing a parameter sweep on both the vertical and in-plane structural parameters: *h*_*α-Si*_, *h*_*b*_, grating period (*Λ*_*g*_) and duty cycle (*DC*). Here, *DC* is defined as the ratio between the length of an unetched silicon segment, *l*_*α-Si*_, and the grating period. The purpose of this parameter sweep is to determine the optimal vertical dimensions (*h*_*α-Si*_, *h*_*b*_) and duty cycle and to identify a range of grating periods that yield high directionality. The diffraction performance of the α-Si grating for the light propagating along the longitudinal (*x*) dimension was optimized using two-dimensional (2D) FDTD simulations of the device cross section depicted in Fig. 1b. The grating is described by the momentum-matching condition^57^:

1 $$ n_{c} \sin \, \Theta_{k} = n_{{{\text{eff}}}} + \frac{k\lambda }{{\Lambda_{g} }} $$

where *n*_c_ is the refractive index of the upper cladding, *Θ*_*k*_ is the diffraction angle (following the sign convention labelled in Fig. 1a), *Λ*_*g*_ is the grating period, *n*_*eff*_ is the effective index of the Floquet-Bloch mode in the grating region, *k* is the diffraction order (in our case, *k* = − 1), and *λ* is the operating wavelength (in vacuum). Figure 2a,b show the dependence of the grating directionality on the buffer oxide thickness, α-Si thickness, *Λ*_*g*_ and *DC*. Based on our 2D FDTD calculations, the optimal thickness of the α-Si layer is 220 nm, yielding directionality exceeding 90% while operating with a single diffraction order. The single-order grating operation was verified by examining the far-field radiation pattern of the uniform grating coupler with optimized directionality. Figure 2c,d show the calculated grating directionality and transmittance as a function of the buffer oxide and BOX thicknesses, respectively. As can be seen from Fig. 2c, the thickness of the buffer oxide layer has a noticeable influence on the grating coupler performance. The peak directionality of 93% is achieved when the radiated field interferes destructively in the Si substrate and constructively in the superstrate medium. On the other hand, the grating transmittance (the residual power at the end of the grating) increases significantly with the buffer oxide thickness. This comes at the expense of a corresponding decrease in the upward radiated power, as depicted in the inset of Fig. 2c. Based on these observations, a buffer oxide thickness of 50 nm was selected to achieve a dual objective: maximizing the directionality and minimizing the transmittance through the grating. Furthermore, the grating directionality also depends on the BOX thickness, as shown in Fig. 2d. For comparison, we also plot in Fig. 2d the directionality for the same grating where the bottom Si substrate is replaced by air. Notably, the directionality peaks for both cases exhibit a distinct out-of-phase relationship with each other. This behavior can be attributed to a partial mirror effect, occurring due to the reflection of the downward radiated beam at the bottom of the BOX layer. The reflection at this bottom interface is combined in-phase with the beam radiated upwards, which then positively contributes to the overall directionality. The fact that the directionality remains high (above 74%) even for the minima of the curve in Fig. 2d highlights that the α-Si/SiN grating structure enhances upward radiation regardless of the BOX thickness. This is an important advantage of our design because the BOX layer thickness is typically not a free parameter, as it is constrained by the platform and offerings of photonic foundry services.

**Figure 3:**
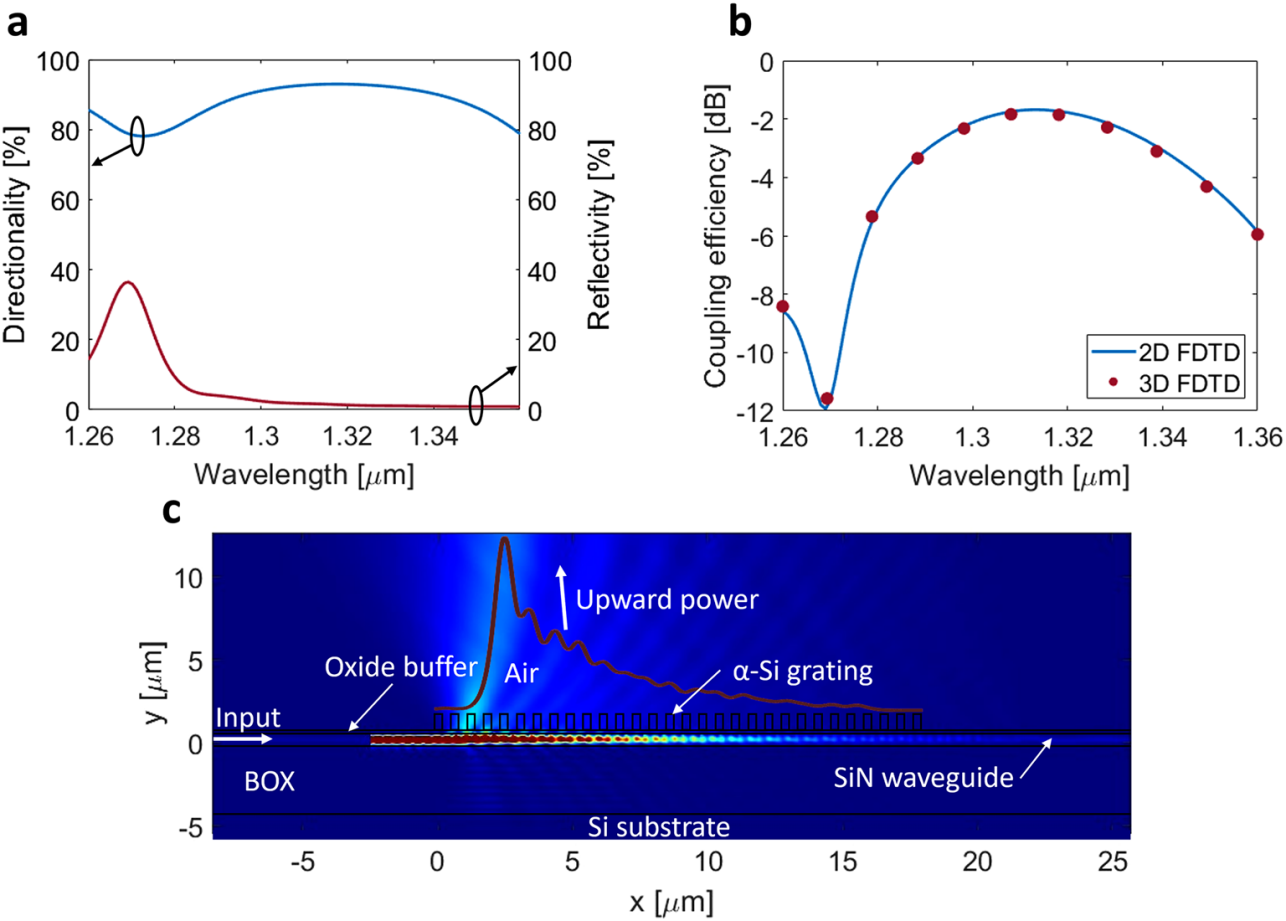
(**a**) Spectral response of grating directionality and back-reflections. (**b**) Coupling efficiency to SMF-28 fiber, as a function of the wavelength. (**c**) Electric field intensity profile in x–y plane (perpendicular to the chip).

Based on the 2D FDTD simulation results shown in Fig. 2b, the optimized grating parameters are: *Λ*_*g*_ = 700 nm and *DC* = 28%, considering a 220-nm-thick α-Si layer and 50-nm-thick buffer oxide. The grating has 31 periods which translates to a length of 21.7 μm, and the optical fiber above the chip is tilted − 4° with respect to the vertical axis (Fig. 1a). For this nominal design, the power radiated towards the optical fiber and the bottom Si substrate is 88% and 7%, respectively, yielding a peak directionality of 93%. At this length, 3% of the power is transmitted through to the end of the grating. The remaining power could be radiated by increasing the length of the grating. However, since the grating is already significantly longer than the mode field diameter of the fiber, adding more periods to the end of the grating would not increase the coupling efficiency because the added radiated power can no longer be directed into the fiber. In the following section we will show how this limitation can be circumvented by leveraging the self-imaging effect, i.e., the grating length is increased while a converging wavefront is imparted on the resulting wide diffracted beam, focalizing it down to the target mode size. Figure 3a shows the spectral dependence of grating directionality and reflectivity. The coupling efficiency, determined by the product of the power radiated upwards and the overlap integral between the radiated field and the Gaussian-like mode of the fiber, is plotted in Fig. 3b. The overlap integral is calculated as:

2 $$ OL = \frac{{\int | E_{g} E_{f}^{*} |^{2} {\text{d}}A}}{{\int {E_{g} } E_{g}^{*} {\text{d}}A\int {E_{f} } E_{f}^{*} {\text{d}}A}} $$

where *E*_*g*_ is the complex electric field radiated by the grating, *E*_*f*_ is the electric field distribution of the optical fiber mode, and the * symbol denotes complex conjugation. At the reference wavelength of 1.31 μm, the calculated fiber-chip coupling efficiency is − 1.7 dB and the back-reflections are as low as 2% (− 17 dB). As shown in Fig. 3b, the 2D and 3D simulations are in excellent agreement. The grating is 13.1-μm-wide to maximize the field overlap between the optical fiber mode and the dominant component of the radiated electric field. The estimated 1-dB and 3-dB coupler bandwidths are 46 nm and 76 nm, respectively. The radiated field of a uniform grating exhibits a decaying exponential profile. As a result, the overlap integral with the SMF-28 fiber mode is estimated to be 77% which limits the coupling efficiency of the grating. Here, the fiber MFD of 9.2 μm was defined as the full width at 1/e^2^ intensity at the nominal wavelength of 1.31 µm^58^. Figure 3c shows a radiation intensity profile of the uniform grating coupler.

### Apodized imaging surface grating coupler

Further enhancement of the coupler performance is achieved by using the grating apodization and self-imaging techniques. The grating strength is apodized to produce a Gaussian profile in the grating’s near-field. Using subwavelength index engineering^38–40,59^, grating teeth with lower refractive indices can be synthesized without the need for additional materials. The corresponding change in refractive index contrast between the SWGs and the grating trenches is used to control the local grating strength. The latter is gradually increased over the first few periods of the coupler, yielding the targeted Gaussian profile. The apodization has the added benefit of reducing the reflectivity of the coupler since the transition between the SiN injection waveguide and the grating is less abrupt. Then, the field is focalized down to the target MFD to overlap with the mode of the optical fiber positioned at the focal plane above the chip. A convergent wavefront in both the longitudinal (*x*-axis) and transversal (*z*-axis) directions is imposed on the diffracted field. Longitudinally, a quadratic phase profile is imparted by chirping the grating period to vary the diffraction angle according to the momentum-matching condition described by Eq. 1. The transverse focusing is implemented through the additional curvature of the grating along the *z*-axis. To begin, the apodized imaging grating is considered in the same simplified 2D topology used to optimize the buffer and α-Si thicknesses.

**Figure 4:**
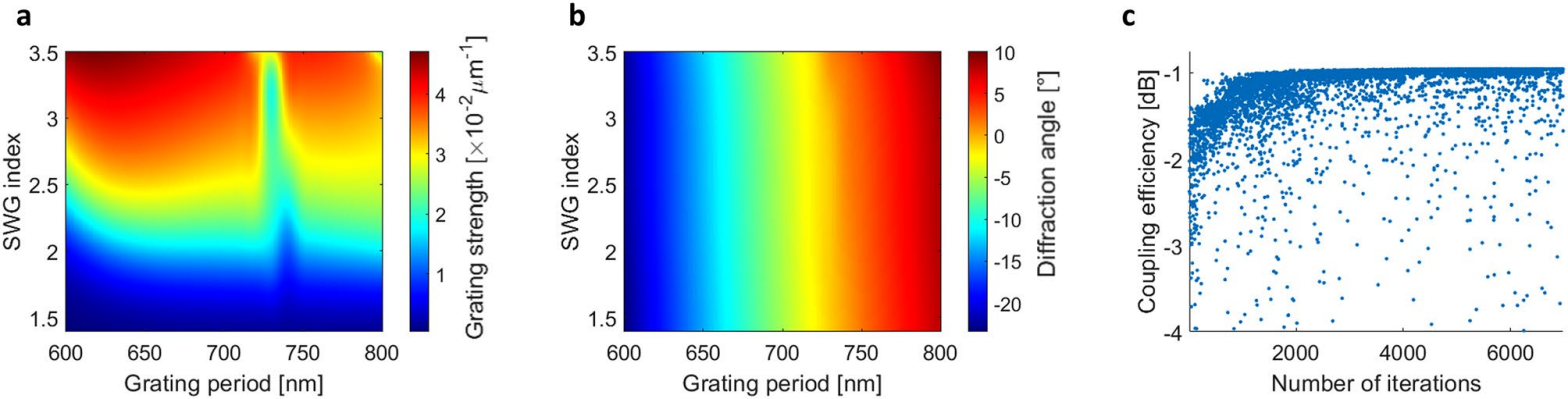
(**a**) Grating strength apodization parameter sweep. (**b**) Diffraction angle as a function of SWG equivalent refractive index and grating period. (**c**) Evolution of coupling efficiency with genetic optimization.

The coupler is comprised of two distinct stages: an apodized section with metamaterial nanostructures, followed by a homogenous grating. In the simplified 2D representation, the two sections are defined by the following criteria: (i) number of apodized periods, (ii) the total number of periods, (iii) the grating strength range, (iv) the diffraction cone (range of diffraction angles for the converging beam) and (v) the vertical chip-to-fiber distance. The genetic algorithm was used to optimize the parameters. The algorithm is seamlessly integrated with Matlab and Ansys Lumerical and is well-suited for problems with discrete variables such as the number of grating periods in our case. In 2D FDTD simulations, the SWGs are represented as a homogenous material with a refractive index between 1 and *n*_*α-Si*_. After optimizing the coupler, the metamaterial nanostructures that synthesize the required SWG indices are determined. The oxide buffer and amorphous silicon layers have thicknesses of 50 nm and 220 nm, respectively, as optimized in the uniform grating coupler design. Figure 4a,b show the apodization maps of the calculated grating strengths and corresponding grating radiation angles, respectively, as functions of equivalent SWG metamaterial index and grating period. At each iteration cycle, the data in Fig. 4a,b is used as a lookup table to assign the structural parameters, i.e., the period and equivalent SWG index, to each grating period, based on the grating strength and diffraction angle ranges set by the genetic algorithm. The coupling efficiency is calculated and is used to determine the next optimization cycle. This process was repeated for several fiber heights to maximize the overlap between the optical fiber mode and radiated grating field while minimizing the effect on the coupler bandwidth due to the dispersive nature of the self-imaging effect^60^. The optimal fiber height was determined to be 55 µm. Figure 4c shows the evolution of the coupling efficiency in the genetic algorithm optimization.

In the optimized design obtained with the genetic algorithm, the SWG equivalent refractive index is increased linearly from 1.45 to *n*_*α-Si*_ within the first 13 periods of the grating. The resulting grating strength increases from 0.002 to 0.047 μm^−1^ as the grating pitch drops from 665 to 639 nm. The apodized section is followed by 27 homogenous periods of uniform grating strength, with the period gradually decreasing from 638 to 603 nm. The diffraction angle changes from − 11° to − 21.8° along the mode propagation direction over the full length of the coupler. The phase engineering focalizes the near-field with an MFD of 16.2 μm down to the target mode size of 9.2 μm at the focal distance of 55 μm above the surface of the grating. The optimized parameters of the apodized imaging grating coupler are summarized in Table 1. The total coupler length is 25.2 μm and the directionality is 88%. The calculated overlap integral reaches 94%, while back-reflections are very low at 0.02% (− 37 dB). The 2D FDTD calculations predict a fiber-to-chip coupling efficiency of − 0.9 dB, with 1-dB and 3-dB optical bandwidths of 34 nm and 59 nm, respectively.

**Table 1:** Optimized parameters of the apodized imaging grating coupler.

	Apodized section	Homogenous section
Number of periods	13	27
Grating pitch	639–665 nm	603–638 nm
Metamaterial index	1.45–*n*_*α-Si*_	Not applicable
Grating strength	0.002 – 0.047 µm^−1^	0.047 µm^−1^
Diffraction angle	− 11° to − 21.8°	
Fiber height	55 µm	

Next, we determine the transverse structure of the device with analytical and numerical 3D analysis. The structural parameters include the width of the injection waveguide, the dimensions of the SiN slab, the grating curvature, and the SWG period and duty cycle. The grating segments are first curved to circumvent the need for an adiabatic taper and collimate the out-coupled beam^58^. We use an input SiN waveguide which is 400-nm-thick and 850-nm-wide to support single-mode operation. The mode of the SiN channel waveguide is injected directly into a wide SiN slab region to allow the mode to expand. In order to achieve focusing effect, an additional curvature factor was imparted on the grating geometry. The coordinates defining the curved grating lines were calculated to equalize the optical path length for the light from the injection waveguide to the tip of the fiber ^25,26^. To this end, we used the coordinates of the grating lines along the central longitudinal axis of the coupler, labelled *C*_*1*_ and *C*_*2*_ in Fig. 1a, as obtained from the 2D optimization. Using the effective index of the slab waveguide region, *n*_*slab*_, the effective index of the grating, *n*_*eff*_, and the coordinate system as in Fig. 1a, the curvature of the grating lines is determined by the optical path relation:

3 $$ n_{{{\text{slab}}}} (\overline{OG}_{1} - \overline{OC}_{1} ) + n_{{{\text{slab}}}} (\overline{{G_{1} G_{2} }} - \overline{{C_{1} C_{2} }} ) + \overline{{G_{2} F}} = \overline{{C_{2} F}} = 0 $$

**Figure 5:**
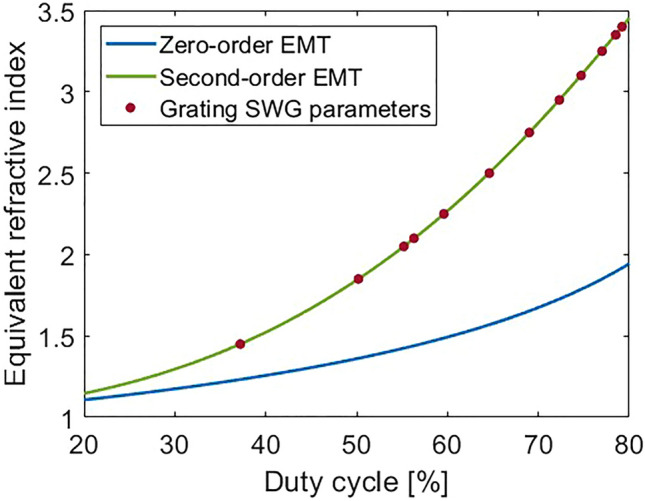
Equivalent refractive index of the SWG metamaterial as a function of the lateral duty cycle for zero- and second-order EMT calculations. The red dots correspond to the SWG parameters used in our design with an SWG period of 600 nm and reference wavelength of 1.31 µm.

The transverse SWG geometry (see Fig. 1c) is determined by the SWG period (*Λ*), and SWG duty cycle, which set the gap width, *w*_*gap*_, and α-Si segment width, *w.* The parameters that synthesize SWG indices obtained with the genetic algorithm optimization were calculated through the effective medium theory (EMT)^44,61^. According to our simulations, an SWG period of 600 nm is sufficiently small to suppress diffraction effects and allows for a minimum feature size of 120 nm, which is compatible with deep UV immersion lithography^62–65^. The second-order EMT was used to calculate the synthesized refractive index^61,66^:

4 $$ n_{ \bot } = n_{ \bot 0} \sqrt {1 + \frac{{\pi^{2} }}{3}\left( {\frac{\Lambda }{\lambda }} \right)^{2} \left( {\frac{w}{\Lambda }} \right)^{2} \left( {1 - \frac{w}{\Lambda }} \right)^{2} \left( {1 - n_{\alpha - Si}^{2} } \right)^{2} \left( {n_{\parallel 0} } \right)^{2} \left( {\frac{{n_{ \bot 0} }}{{n_{\alpha - Si} }}} \right)^{4} } $$

where *w* is the width of the α-Si SWG segments, *Λ* is the SWG period, *n*_α-Si_ is the refractive index of the amorphous silicon, *λ* is the wavelength, and:

5 $$ n_{\parallel 0}^{2} = \frac{w}{\Lambda }n_{\alpha - Si}^{2} + \left( {1 - \frac{w}{\Lambda }} \right) $$

6 $$ \frac{1}{{n_{ \bot 0}^{2} }} = \frac{w}{\Lambda }\frac{1}{{n_{\alpha - Si}^{2} }} + \left( {1 - \frac{w}{\Lambda }} \right) $$

are the zero-order EMT equations for light polarized in the direction parallel and perpendicular to the SWG interfaces, respectively. A comparison of the second- and zero-order EMT models as well as the parameters used in our optimized design are shown in Fig. 5. The etched gaps are varied from 380 to 138 nm over the first 12 periods. The final period with the maximum grating strength consists of a solid α-Si segment. Accounting for the curvature of the grating lines, the overall footprint of the grating coupler is 26.8 × 32.7 µm^2^.

**Figure 6:**
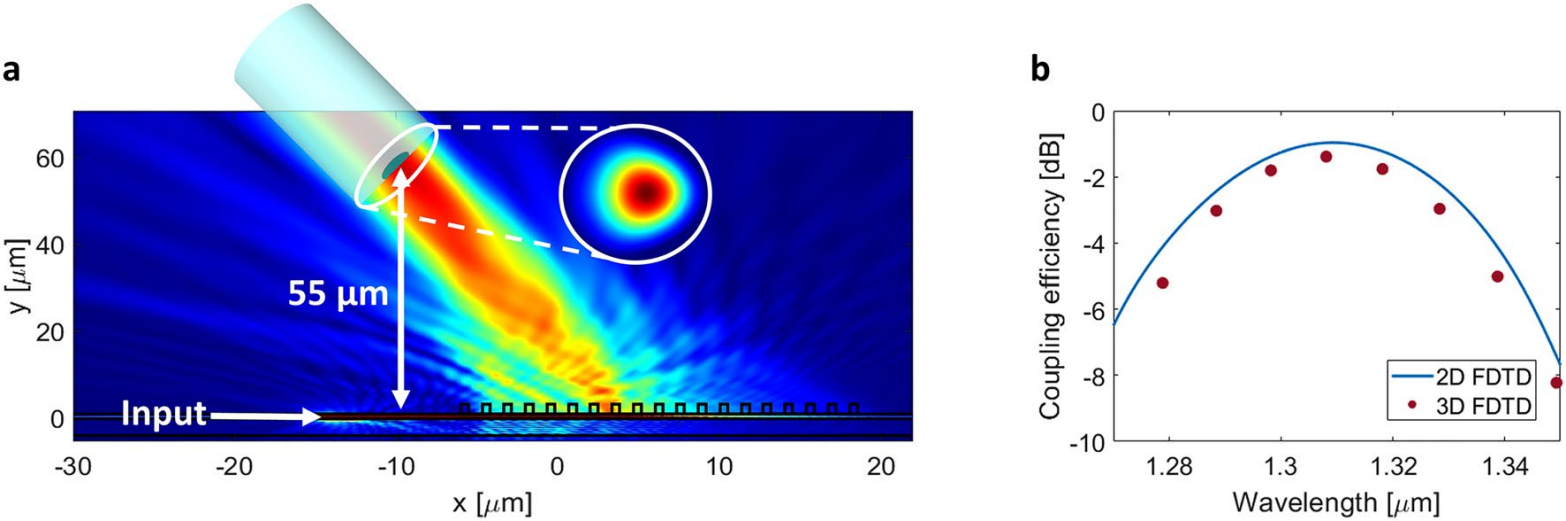
(**a**) Radiation intensity profile for the optimized apodized imaging surface grating coupler. (**b**) Coupling efficiency to an SMF-28 fiber at the 55 µm focal length.

The radiation intensity profile of our optimized design and its spectral performance are shown in Fig. 6a,b, respectively. Here, the input light is injected from the SiN waveguide and then radiated out by the α-Si grating and focused onto the optical fiber. A peak coupling efficiency of − 1.3 dB at the 1.31 μm wavelength with a directionality of 85% and mode field overlap of 90% is predicted by 3D FDTD simulations. The corresponding 1-dB and 3-dB coupler bandwidths are 31 nm and 52 nm, respectively. The small discrepancy between 2D and 3D simulations is likely caused by a mismatch between the desired equivalent refractive index of the individual SWG metamaterial segments and the actual engineered refractive index. The EMT is strictly valid for infinitely stratified media and does not consider the effect of waveguide confinement.

## Conclusion

In summary, we have presented a novel design of fiber-chip surface grating coupler, implemented in a hybrid α-Si/SiN photonic platform operating at the 1.31 μm wavelength. The coupling efficiency is enhanced using a combination of a high-index amorphous silicon grating, subwavelength metamaterial apodization, and the self-imagine effect. Our design strategy simultaneously increases the directionality of the grating by breaking the vertical symmetry while at the same time optimizes the overlap between the diffracted field and the optical fiber mode by grating apodization and self-imaging effect. The later is used to focalize the radiated field onto an SMF-28 optical fiber positioned at a focal point about 55 µm above the chip. The grating has a compact footprint of 26.8 × 32.7 µm^2^ and can be fabricated with a single-etch step fabrication process. The coupler’s vertical dimensions and apodization parameters were optimized using genetic algorithm-assisted 2D FDTD simulations of the longitudinal cross section of the device. The transverse structure was then designed and verified by rigorous 3D FDTD simulations. Our optimized grating coupler design exhibits a peak coupling efficiency of − 1.3 dB over 1-dB and 3-dB bandwidths of 31 nm and 52 nm, respectively. This outstanding design performance is achieved while maintaining a minimum feature size above 120 nm, compatible with deep-UV lithography. We believe this work constitutes an important advance in the development of efficient silicon nitride based off-chip coupling interfaces for O-band applications of integrated photonic circuits, including datacom interconnects and quantum photonics.

## Data Availability

Data generated or analyzed in this study may be obtained from the contact author upon reasonable request.
